# Metastasis of Renal Cell Carcinoma Causing Significant Facial Asymmetry

**DOI:** 10.1155/2019/6840873

**Published:** 2019-11-03

**Authors:** Rafael Netto, Silas Antonio Juvencio de Freitas Filho, Wladimir Cortezzi, Flávio Merly, Vitor Marcello de Andrade, Fábio Ramôa Pires

**Affiliations:** ^1^Stomatology Service, Department of Oral Diagnosis and Pathology, School of Dentistry, Federal University of Rio de Janeiro, Rio de Janeiro, RJ, Brazil; ^2^Department of Surgery, Stomatology, Pathology and Radiology (Area of Pathology), Bauru School of Dentistry, University of São Paulo, Bauru, SP, Brazil; ^3^Oral and Maxillofacial Surgery Service, Federal Hospital of State Servers, Brazilian Government, Rio de Janeiro, RJ, Brazil; ^4^Department of Oral Pathology, School of Dentistry, State University of Rio de Janeiro, Rio de Janeiro, RJ, Brazil

## Abstract

The occurrence of metastatic tumors in the orofacial region is rare and may represent the first clinical manifestation of occult malignant disease. An orofacial lesion diagnosed as a metastatic tumor from a renal cell carcinoma in a 68-year-old man is reported. This metastatic tumor caused significant facial asymmetry involving the parotid gland and mandible regions, and the patient died four months after diagnosis. Here, we discuss the clinical aspects, the diagnostic approach, and the importance of early diagnosis to obtain a better response to treatment and provide longer survival time.

## 1. Introduction

Oral metastatic tumors are uncommon lesions that can occur at any age and in both genders, which represent the systemic spread of the disease or initial presentation of an unknown malignancy at a distant place [1].

Metastatic lesions to the oral cavity can occur in the jawbones and soft tissues, including salivary glands [2–5]. Oral metastases arise most commonly from the breast, liver, and thyroid for women [2]. In men, the most common origin of oral metastatic tumors is from the lung, kidney, liver, and prostate [2, 4].

The clinical and radiological aspects of metastatic tumors are varied, making diagnosis and therapeutic management challenging [6], so it is highly relevant to investigate the patient's medical history. The lesions may be asymptomatic or painful and may cause paresthesia, tooth mobility, regional lymphadenopathy, local swelling, exophytic growth, modification of the normal aspects of the oral mucosa, and still pathologic fracture in osseous lesions [7].

Histopathological findings are essential and generally reflect the pattern of primary tumors [8]. Due to the rarity of these metastatic tumors in the maxillofacial region and the challenging diagnosis, the immunohistochemical expression is an auxiliary diagnostic tool [7, 8].

Herein, we report and discuss a case of oral metastasis with significant facial asymmetry involving the parotid gland and mandible regions, resulting from a primary renal cell carcinoma.

## 2. Case Presentation

A 68-year-old male patient was referred by the public health service of his city for specialized care to the Oral and Maxillofacial Surgery Service of the Federal Hospital of Rio de Janeiro State Servers/Brazil for the evaluation of volumetric increase on the right side of the face. Medical history has reported systemic hypertension and gastritis, both conditions under drug control. He also reported having had surgery for an appendectomy at 25 years of age and denied allergies. The patient reported frequent use of alcohol and smoking for 35 years and having stopped smoking 4 years ago. The patient reported a progressive and painless growth of lesion in the genian region in the last four months.

**Figure 1:**
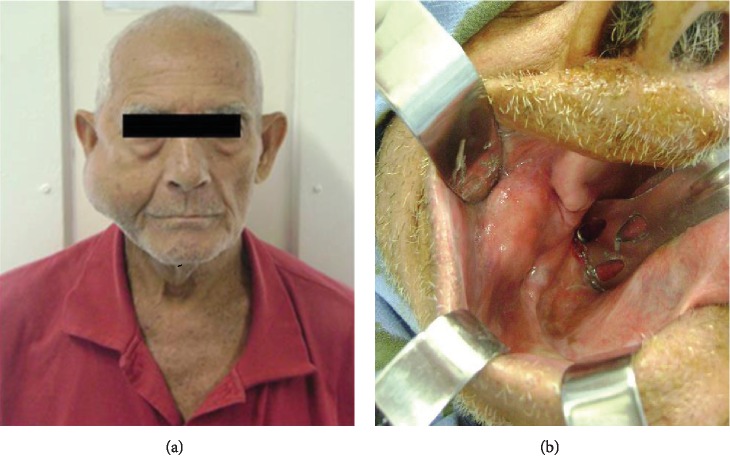
(a) Extraoral clinical aspect causing significant facial asymmetry. (b) Intraoral clinical aspect shows a slight elevation in the jugal mucosa.

Extraoral examination revealed a tumor-like lesion, firm consistency, well-defined contours, and significant expansion near the parotid region (Figure 1(a)). On intraoral examination, a slight elevation in the ipsilateral jugal mucosa was noted, corresponding to the expansion of the medial growth tumor (Figure 1(b)).

**Figure 2:**
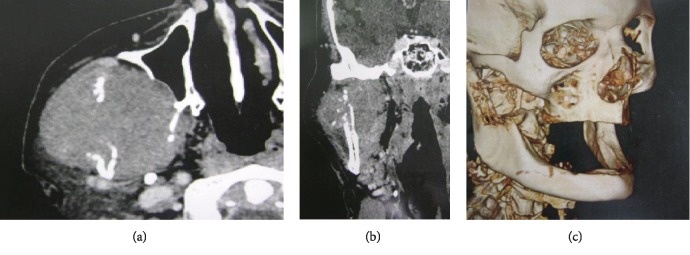
(a) Axial CT image reveals an expansive lesion in the soft tissue of mixed aspect and regular contours. (b) Coronal CT image shows hyperdense foci among the lesions. (c) 3D reconstruction of CT scan reveals mandibular ramus lytic lesions.

For an incisional biopsy, computed tomography (CT) images of the face and preoperative laboratory tests were requested. The results showed normal serum levels of most of the requested blood fractions, except for slightly elevated urea and creatinine. The axial CT image (Figure 2(a)) revealed an expansive lesion (approximately 6.0 × 6.0 cm) in the soft tissue of the mixed aspect and regular contours. In the coronal CT image, hyperdense foci of the lesion were observed (Figure 2(b)), suggestive of calcification or bone lysis. In the three-dimensional reconstruction of the tomography (Figure 2(c)), bone destruction in the mandibular branch was evidenced by possible tumor compression.

**Figure 3:**
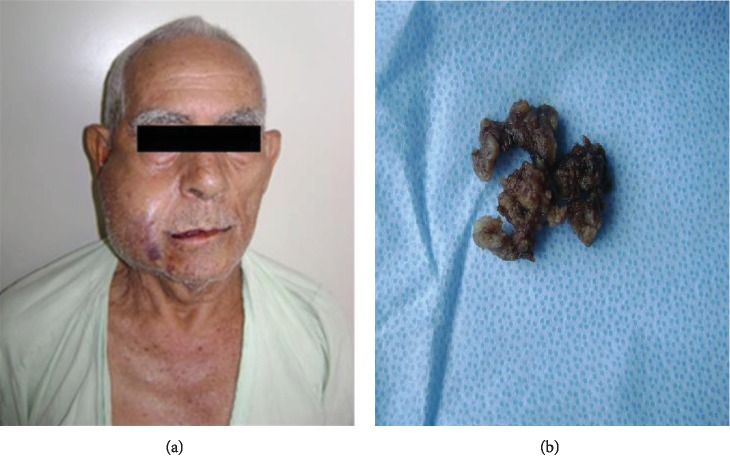
(a) Clinical aspect 72 hours after an intraoral incisional biopsy showing significant expansion of the lesion. (b) Surgical specimen.

An intraoral incisional biopsy was performed. In the first 72 hours after the biopsy, the patient developed significant local edema and volumetric expansion of the lesion, which became inaccurate (Figure 3(a)). On gross examination, three fragments of smooth brown tissue were observed (Figure 3(b)), which were sent for histopathological analysis. After anamnesis and clinical and radiological examinations, our diagnostic hypotheses were Warthin's tumor, pleomorphic adenoma, mucoepidermoid carcinoma, and soft tissue sarcoma.

**Figure 4:**
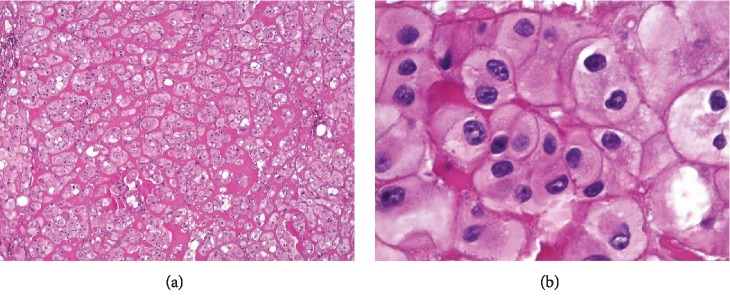
Microscopic analysis: (a) large neoplastic cells often eosinophilic and pale with reticulated cytoplasm and a centralized oval nucleus; (b) neoplastic cells with a centralized oval nucleus, perinuclear halo, and prominent nucleoli (H and E staining: (a) 100x and (b) 400x).

Microscopically, large neoplastic cells often eosinophilic and pale, sometimes clear, with reticulated cytoplasm, centralized oval nucleus and perinuclear halo, and prominent nucleoli were observed (Figure 4). The diagnosis was strongly suggestive of oral metastasis of chromophobe renal cell carcinoma.

**Figure 5:**
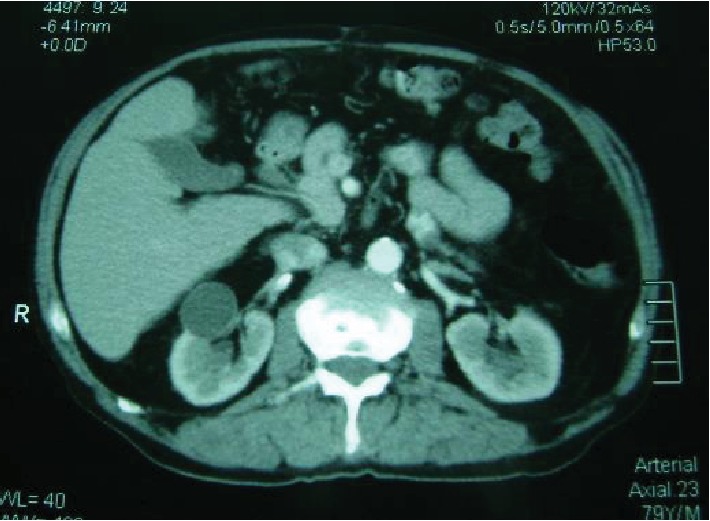
Axial CT image of the abdomen reveals a lesion involving the right kidney cortex and parenchyma.

The patient underwent primary CT scan of the abdomen, which showed the presence of lesion involving the right kidney cortex and parenchyma (Figure 5). The patient was referred to the Referral Oncology Service, where renal cell carcinoma was effectively diagnosed, and then began treatment with chemotherapy for kidney tumor and radiotherapy for orofacial metastatic tumor. The patient died four months after starting treatment.

## 3. Discussion

Metastases to the oral cavity or head and neck region are extremely rare conditions [7, 9]. Metastatic tumors account for less than 1% of all oral malignancies [7, 10]. Such metastases can affect men and women equally and at any age [11]. According to Hirshberg et al. [4], the kidney is the third most common primary site for oral metastases, although there is underreporting [8]. The incidence of oral metastases and their origins may vary according to the study population [4, 7, 11].

As shown in our case, oral lesions have been reported among the first manifestations of an undiagnosed primary malignant disease [7, 11] or may still be indicative of terminal disease [1]. Initially, our patient had no signs or symptoms for suspected metastatic tumor. Among the main signs and symptoms, pain and swelling of the region with a metastatic tumor have been observed in most patients [2]. Clinically, orofacial metastases of renal cell carcinoma may show the following characteristics: submucosal or jaw mass, jaw or oral pain, lip numbness, gingival swelling, or even an extensive bilobed mass [1]. Other signs may be noted as bleeding, tooth mobility, trismus, paraesthesia, and paralysis [2, 11]. When bone tissue is involved by metastatic tumors, lytic lesions are often observed [4]. Despite the significant size of the lesion, our patient had no painful symptoms, and surprisingly lytic lesions in the mandibular branch were found on radiological examinations, although the lesion mainly involved the maxillary region. In addition, the use of technologies such as PET-CT collaborates in identifying primary or metastatic skeletal tumors [12].

Initially, the location, size, and limits of the lesion led to the clinical hypothesis of a primary neoplastic lesion in the parotid gland, mainly Warthin's tumor due to cigarette use. However, mandibular bone resorption was indicative of aggressiveness of the lesion. After the surgical intervention for an incisional biopsy and worsening of the clinical picture, our team began to consider a malignant or metastatic tumor. However, for cases with a history of cancer, the professional should evaluate the patients' complaint and reflect on the inclusion of metastatic disease among the diagnostic hypotheses [13, 14]. Oral metastases of renal cell carcinoma may show clinical features similar to those of aggressive malignant tumors [15]. For cases with a suspected parotid gland tumor, the surgeon may perform a fine-needle aspiration biopsy, avoiding possible discomfort and complications from an incisional biopsy [8].

In the study by Liu et al. [7], all cases of oral metastasis from CCR had no previous diagnosis, showing that this tumor is insidious in nature. For tumor lesions in the orofacial region, histopathological analysis is mandatory to distinguish the neoplasia and then define the treatment plan and outline the patient's prognosis [16]. Often, the definitive diagnosis with no previous history of cancer is challenging to the pathologist. Thus, an immunohistochemical panel with limited or broader molecules may be useful in establishing the origin of the tumor and the definitive diagnosis [7, 8, 10, 12]. In the present case, due to the experience of the pathological anatomy service with head and neck metastatic tumors associated with the infrastructure provided by the hospital, our patient was submitted to the investigation of primary renal neoplastic injury immediately after microscopic analysis. Also, for cases similar to ours, the immunohistochemistry has not been a frequent technique in establishing a diagnosis [1].

Since metastases reflect the primary tumor pattern [7], microscopic analysis is essential for predicting the neoplasia's biological behavior. Although most of these tumors are clear cell type, a thorough evaluation of other more frequent patterns such as papillary and chromophobe is relevant for understanding the disease. Although the chromophobe pattern of renal cell carcinoma is associated with low mortality, in our case, the late diagnosis was relevant in the outcome of the disease [17]. In addition, Pires et al. [15] affirm the importance of immunohistochemical differentiation between metastatic renal cell carcinoma and mucoepidermoid carcinoma in case of overlapping of histopathological features.

According to Owosho et al. [1], adult patients with oral metastases have a worse prognosis compared to pediatric patients, and in adults, oral soft tissue involvement leads to a shorter survival compared to patients with mandibular bone involvement. For cases similar to ours, the treatment modalities are surgical resection, radiotherapy, and chemotherapy [14], and there may be variation among metastatic tumors of renal cell carcinomas [1]. Moreover, the use of antiangiogenic agents and genome sequencing are promising therapeutic modalities [1]. Our elderly patient with advanced metastatic disease did not respond to treatment and had a shorter survival time than that found in the study by Hirshberg et al. [4]. The conservative and palliative treatment has been recommended for cases with worse prognosis, while the option for surgical removal is restricted to cases with less advanced disease [14].

In summary, the occurrence of metastatic tumors in the orofacial region is rare and may represent the first clinical manifestation of occult malignant disease. These lesions are very serious and have a poor prognosis. Wherever possible, early diagnosis should be performed to obtain a satisfactory response to treatment and provide better patient survival.
